# Continuing professional development – Radiation therapy

**DOI:** 10.1002/jmrs.659

**Published:** 2023-02-10

Maximise your CPD by reading the following selected article and answer the five questions. Please remember to self‐claim your CPD and retain your supporting evidence. Answers will be available via the QR code and online at www.asmirt.org/news-and-publications/jmrs, as well as published in JMRS – Volume 70, Issue 4 December 2023.

## Radiation Therapy – Original Article

### Volumetric modulated arc therapy (VMAT) comparison to 3D‐conformal technique in lung stereotactic ablative radiotherapy (SABR)

Mark F , Alnsour A , Penfold SN , Esterman A , Keys R , Le H . (2023) J Med Radiat Sci. 10.1002/jmrs.634 PMC997766436424510

1. Which of the following statements regarding radiation pneumonitis is *most* correct?
  - Radiation pneumonitis is a minor side effect of stereotactic ablative radiotherapy (SABR) treatment
  - Radiation pneumonitis is not a dose‐limiting toxicity in the treatment of lung tumours
  - Radiation pneumonitis is a rare side effect that only occurs in patients with a pre‐existing lung condition
  - Radiation pneumonitis increased risk is unlikely to be associated with volumetric modulated arc therapy (VMAT) treatment
2. What is the benefit of volumetric modulated arc therapy (VMAT) compared with the standard 3D‐conformal technique?
  - Quicker planning time as minimal contours are required for treatment planning
  - Reduced staff workload with fewer quality assurance checks required
  - The dose per fraction can be escalated resulting in less treatment fractions
  - Advanced radiation dose shaping can be achieved without compromising on treatment time
3. What does the conformity index (CI) measure?
  - How well two different Radiation Therapists will create a similar treatment plan for any given patient
  - The inter‐variability of different Radiation Oncologists tumour contouring
  - The quality of treatment plans and represents the relationship between isodose distributions and target volume
  - The dose drop‐off distance from the 100% to 50% isodose lines outside the planning treatment volume (PTV)
4. In this study, what SABR dose regime was utilised?
  - 45 Gy/3 fractions
  - 48 Gy/4 fractions
  - 60 Gy/3 fractions
  - 60 Gy/4 fractions
5. Which of the following was identified as a limitation of this study?
  - The sample size was too small to produce reliable results, more data sets are required to increase the statistical power
  - This study included VMAT and 3D‐CRT plans for all patients with inoperable stage I‐II non‐small cell lung cancer (NSCLC), resulting in greatly varying PTV volumes
  - The treatment planners were quite inexperienced with SABR planning, hence results from this study may not be reliable
  - The participants in this study had such advanced disease resulting in significantly large PTV volumes, hence results from this study cannot be generalised across all populations

## Answers


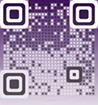


Scan this QR code to find the answers, or visit www.asmirt.org/news-and-publications/jmrs
